# Patterns of Suicide in the Context of COVID-19: Evidence From Three Australian States

**DOI:** 10.3389/fpsyt.2021.797601

**Published:** 2021-11-30

**Authors:** Angela Clapperton, Matthew John Spittal, Jeremy Dwyer, Andrew Garrett, Kairi Kõlves, Stuart Leske, Ciara Millar, Bronwen Edwards, Victor Stojcevski, David Robert Crompton, Jane Pirkis

**Affiliations:** ^1^Melbourne School of Population and Global Health, The University of Melbourne, Carlton, VIC, Australia; ^2^Coroners Prevention Unit, Coroners Court of Victoria, Melbourne, VIC, Australia; ^3^Coronial Division, Magistrates Court of Tasmania, Hobart, TAS, Australia; ^4^Australian Institute for Suicide Research and Prevention, School of Applied Psychology, Griffith University, Brisbane, QLD, Australia; ^5^Roses in the Ocean, Newstead, QLD, Australia

**Keywords:** mental health, suicide, COVID-19, epidemiology, risk factors

## Abstract

**Aims:** We aimed to determine whether there has been a change in the number of suicides occurring in three Australian states overall, and in age and sex subgroups, since the COVID-19 pandemic began, and to see if certain risk factors for suicide have become more prominent as likely underlying contributing factors for suicide.

**Method:** Using real-time data from three state-based suicide registers, we ran multiple unadjusted and adjusted interrupted time series analyses to see if trends in monthly suicide counts changed after the pandemic began and whether there had been an increase in suicides where relationship breakdown, financial stressors, unemployment and homelessness were recorded.

**Results:** Compared with the period before COVID-19, during the COVID-19 period there was no change in the number of suicides overall, or in any stratum-specific estimates except one. The exception was an increase in the number of young males who died by suicide in the COVID-19 period (adjusted RR 1.89 [95% CI 1.11–3.23]).

The unadjusted analysis showed significant differences in suicide in the context of unemployment and relationship breakdown during the COVID-19 compared to the pre-COVID-19 period. Analysis showed an increase in the number of suicides occurring in the context of unemployment in the COVID-19 period (unadjusted RR 1.53 [95% CI 1.18–1.96]). In contrast, there was a decrease in the number of suicides occurring in the context of relationship breakdown in the COVID-19 period (unadjusted RR 0.82 [95% CI 0.67–0.99]). However, no significant changes were identified when the models were adjusted for possible over-dispersion, seasonality and non-linear trend.

**Conclusion:** Although our analysis found no evidence of an overall increase in suicides after the pandemic began, the picture is complex. The identified increase in suicide in young men indicates that the impact of the pandemic is likely unevenly distributed across populations. The increase in suicides in the context of unemployment reinforces the vital need for mitigation measures during COVID-19, and for ongoing monitoring of suicide as the pandemic continues.

## Introduction

The coronavirus disease 2019 (COVID-19) pandemic has had damaging health, social and economic impacts across the world. In some countries the health impacts were predominately the large number of deaths directly resulting from COVID-19 (1). In countries like Australia, where COVID-19 cases and deaths have been fewer, the health impacts are likely due to the consequences of social distancing and stay-at-home orders (2). There has been considerable concern about the mental health of populations during this time, particularly concerns that suicides might increase as a consequence of the pandemic (3–5). The all-encompassing nature of the pandemic, and in particular, the stay-at-home orders designed to reduce the spread of COVID-19, may mean that people who already experience poor mental health may experience even worse outcomes, and others who have previously experienced good mental health may develop new mental health problems associated with the stress of the pandemic (4). In addition, people may be unable, or less likely to access mental health care, due to barriers associated with stay-at-home directives and general fear of contracting COVID-19. To date, most international and Australian based research has found no evidence of an overall increase in suicides in the initial months of the pandemic (6–8). However, experts have noted that we need to remain vigilant and that increases in suicide may still occur (2, 9). Recent findings from Japan suggest this caution is warranted; after an initial reduction in suicide in that country, there was a subsequent increase (10, 11).

In addition to monitoring overall trends in suicide as the pandemic continues, it is essential to consider whether patterns may vary for different subgroups in the population (9). Monitoring suicide numbers for different demographic subgroups (e.g., males and females in different age groups) is important. However, it is also important to track whether specific social determinants of suicide are increasingly implicated in suicides during the pandemic. The flow-on effects from the economic consequences of the pandemic such as financial problems, unemployment and homelessness are a major concern, given the global economic crisis of 2008 was associated with an increase in suicide in several countries (12). Another risk factor for suicide that the pandemic may heighten is relationship breakdown. Research suggests that couples who experience elevated stress, as is likely for many reasons during COVID-19, interact using less adaptive relationship processes and are therefore at greater risk for relationship deterioration (13).

In Australia, research from Queensland using the interim Queensland Suicide Register (iQSR) found no absolute or relative increases in four motives for suspected suicides, namely recent unemployment, financial problems, relationship breakdown, or domestic violence in the initial months of the pandemic (7). Similarly, a Victorian study using the Victorian Suicide Register (VSR), found no increase in the frequency of suicides following the onset of COVID-19. However, the study identified 60 instances of “COVID-linked suicides” (9.5% of the total suicides included in the study) (8). The current study complements these studies by pooling data from the iQSR, the VSR, and the Tasmanian Suicide Register (TSR), which typically account for nearly half of all suicides occurring annually in Australia.

The aims of this study were two-fold: (1) to determine whether there has been a change in the number of suicides occurring overall and in age and sex subgroups since the COVID-19 pandemic began; and (2) to determine whether particular risk factors for suicide (namely relationship breakdown, financial stressors, unemployment, and homelessness) have become more prominent as likely underlying contributing factors for suicide during the pandemic.

## Method

### Study Design

Using real-time suicide data from the iQSR, the VSR and the TSR that is largely drawn from police reports (see below), we ran multiple interrupted time-series analyses (ITSA) to ascertain whether trends in monthly suicide counts changed after the pandemic began and whether there had been an increase in suicides where relationship breakdown, financial stressors, unemployment and homelessness were recorded.

### Data Sources and Inclusion Criteria

We pooled data from the iQSR, the VSR and the TSR for the period from 1st January 2017 to 31st August 2020. The fact that the data sources for this study are real-time registers means that the suicides registered within them are “suspected” based on coders' judgements (which in turn are based largely on initial police evidence) and not yet confirmed by the coroner.

Victorian Health Authorities confirmed the first case of COVID-19 in Australia on the 25th January 2020, and Queensland (and New South Wales) confirmed cases in the following days (13, 14). Therefore, we chose 1st February 2020 as the beginning date of our COVID-19 period and included data on suspected suicides occurring in the 37 months before (1st January 2017 to 31st January 2020) and the seven months after (1st February 2020–31st August 2020). Variables of interest were age group and sex of the deceased, the month of death, and evidence of relationship breakdown, financial stressor, unemployment or homelessness.

#### Interim Queensland Suicide Register (IQSR)

The sole data source for the iQSR is the Form 1 police report of a death to a coroner. These reports inform coroners of the circumstances of the death and characteristics of decedents, to assist forensic pathologists and coroners in determining the cause of death and potential intent of the deceased. The Form 1 police reports are completed by a police officer soon after a death, following an interview with the deceased's next-of-kin or other available people. The iQSR methodology is discussed in detail elsewhere (15). For this study we took the responses to the question “Is there any possible motive/trigger for the suicide?” and coded the presence of relationship breakdown, financial stressors and unemployment. We used the variable “residency” to code for evidence of homelessness.

#### Victorian Suicide Register (VSR)

The VSR includes the full text of the summary of circumstances that Victoria Police submit when initially reporting a death to the Coroners Court of Victoria. The summary of circumstances is an unstructured narrative prepared in the hours after the death or discovery of body and includes whatever relevant information the reporting police officer can ascertain based on attendance at the scene of death and speaking with witnesses. The detail and accuracy of information contained therein largely depends on what can be established at the time. Some summaries include extensive accounts of the events leading up to death recounted by family members, acquaintances and treating medical practitioners, and through suicide notes found at the scene. In other cases the summary of circumstances may be little more than a description of the scene of death. For this study, we coded the presence of the four risk factors of interest if there was any suggestion in the police summary that those factors may have been implicated in the suicide.

#### Tasmanian Suicide Register (TSR)

Notification of suspected suicides for the TSR is by the police report of death for the coroner. Police officers who first attend the death complete this report. It is based on a mix of structured items (e.g., date and time of death, socio-demographic information) and an additional unstructured narrative of police circumstances generated following interviews with witnesses or the senior next-of-kin or both. Suicides are classified using these initial interviews, any prior statements by the deceased to family friends, notes/letters outlining intent, and known previous suicide attempts. Each of these is recorded on the police report of death. To code the presence of stressors in each suicide, we used certain items on the police report, namely the presence of possible motives/triggers for the suicide (specifically relationship breakdown, financial problems and unemployment). We used the variable “residence status” to determine whether the individual was homeless.

### Data Analysis

We combined data from the three registers into a single dataset that aggregated data at the monthly level for six different strata (men and women aged under 25 years, 25–64 years and 65 years and older). We used these data to estimate any change in the number of suicides during the pandemic compared with the period before the pandemic, as defined above.

We explored whether there was a change in the number of suicides per month in unadjusted and adjusted analyses. In our unadjusted analysis, we compared the average number of suicides per month in the pre-COVID-19 period (the number of suicides divided by the number of pre-COVID months) to the average number of suicides per month during the first months of the pandemic (calculated the same way as for the pre-pandemic period). We did this by calculating a rate ratio, defined as the ratio of these two averages. We did this for all suicides, for age-sex strata, and for the age-sex strata of the four different risk factors (i.e., relationship breakdown, unemployment, financial stressors, and homelessness).

In our adjusted analyses, we calculated the rate ratio using ITSA by fitting Poisson regression models to the monthly data. Our model included a binary variable for the pandemic (coded 0 in the pre-COVID-19 period and 1 during the pandemic), and non-linear terms for time, including seasonality. The ITS model was therefore:


$$\begin{array}{l} \log\left(y_{t}\right)\;=\;\beta_{0}+\beta_{1}x_{1}+f\left(time\right) \end{array}$$

where *x*_1_ was a binary coded variable equal to 1 if the observation is during the pandemic period and 0 otherwise, *f*(*time*) was a function for time. The exponential of the parameter β_1_ was interpreted as the rate ratio of interest (i.e., $e^{\beta_{1}}\;=\;RR$). The function for time comprised two components: long-term time trends (using fractional polynomials) and short-term trends (using Fourier terms, i.e., Sine and Cosine pairs). Overdispersion was addressed by using a scaling parameter set to the estimated Pearson chi square statistic divided by the residual degrees of freedom. Adjusted analyses were run for all suicides, for age-sex strata, and for the age-sex strata of the four different risk factors. We conducted all analyses using Stata software (version 16.0).

## Results

Over the entire study period there were 5,791 suicides recorded across Queensland, Victoria, and Tasmania (Table 1). Males accounted for 75.4% of these suicides (*n* = 4,366) and most occurred among people aged 25–64 years (70.5%, *n* = 4,080).

**Table 1:** Counts of suspected suicides in each period (before and during COVID-19), by age group and sex.

	**Total**		**Pre-COVID-19** **(37 months)**		**COVID-19** **(7 months)**	
	***n***	**% of all suicides**	***n***	**% of all suicides**	***n***	**% of all suicides**
All	5,791	100.0	4,878	100.0	913	100.0
<25 years	833	14.4	685	14.0	148	16.2
25–64 years	4,080	70.5	3,461	71.0	619	67.8
65+ years	878	15.2	732	15.0	146	16.0
Males	4,366	75.4	3,676	75.4	690	75.6
<25 years	617	10.7	504	10.3	113	12.4
25–64 years	3,104	53.6	2,633	54.0	471	51.6
65+ years	645	11.1	539	11.0	106	11.6
Females	1,425	24.6	1,202	24.6	223	24.4
* <25 years*	216	3.7	181	3.7	35	3.8
*25–64 years*	976	16.9	828	17.0	148	16.2
*65+ years*	233	4.0	193	4.0	40	4.4

In the pre-COVID-19 period there were 4,878 suicides recorded, or an average of 132 suicides per month. In the COVID-19 period there were 913 suicides recorded, or an average of 130 per month. Males accounted for approximately three-quarters of suicides in both the pre-COVID-19 period and the COVID-19 period (75.4 and 75.6%, respectively). People aged 25–64 years accounted for 70.5% of suicides overall, 71.0% in the pre-COVID-19 period and 67.8% in the COVID-19 period.

Of the four risk factors for suicide examined in this study, relationship breakdown was the most frequently recorded by the police (Table 2). There were 1,336 cases of suicide for which relationship breakdown was recorded, 23.5% in the pre-COVID-19 period and 20.9% of cases in the COVID-19 period. There were 442 cases of suicide for which the presence of financial stressors was recorded (7.6% in the pre-COVID-19 period and 8.0% in the COVID-19 period), 361 for which unemployment was recorded (5.7% in the pre-COVID-19 period and 8.9% in the COVID-19 period), and 90 for which homelessness was recorded (1.5% in the pre-COVID-19 period and 1.6% in the COVID-19 period).

**Table 2:** Counts of suspected suicides in each period (before and during COVID-19), by the presence of risk factors.

	**Total**		**Pre-COVID-19** **(37 months)**		**COVID-19** **(7 months)**	
	***n***	**% of all suicides**	***n***	**% of all suicides**	**n**	**% of all suicides**
RISK FACTORS						
Relationship breakdown	1,336	23.1	1,145	23.5	191	20.9
Financial stressors	442	7.6	369	7.6	73	8.0
Unemployment	361	6.2	280	5.7	81	8.9
Homelessness	90	1.6	75	1.5	15	1.6

### Changes Over Time

Table 3 shows the observed number of suicides in the pre-COVID-19 period and the COVID-19 period, the mean number of suicides per month in each period, and the unadjusted and adjusted rate ratios (RRs). Compared with the pre-COVID-19 period, during the COVID-19 period there was no change in the number of suicides overall (unadjusted RR 0.99 [95% CI 0.92–1.06]; adjusted RR 1.10 [95% CI 0.93–1.32]), or in any stratum-specific estimates except one. The exception was an increase in the number of young males dying by suicide in the COVID-19 period in the adjusted model (adjusted RR 1.89 [95% CI 1.11–3.23]).

**Table 3:** Unadjusted and adjusted rate ratios of suspected suicides in the COVID-19 period based on the trends in the pre-COVID-19 period.

	**Number of suicides**		**Mean suicides (per month)**		**Unadjusted rate ratio**	**Adjusted rate ratio**
	**Pre-COVID-19 (37 months)**	**COVID-19 (7 months)**	**Pre-COVID-19**	**COVID-19**	**RR (CI)**	**RR (CI)**
All	4,878	913	131.84	130.43	0.99 (0.92, 1.06)	1.10 (0.93, 1.32)
Males	3,676	690	99.35	98.57	0.99 (0.91, 1.08)	1.12 (0.90, 1.41)
<25 years	504	113	13.62	16.14	1.19 (0.96, 1.46)	1.89 (1.11, 3.23)^**^
25–64 years	2,633	471	71.16	67.29	0.95 (0.86, 1.04)	1.01 (0.83, 1.22)
65+ years	539	106	14.57	15.14	1.04 (0.84, 1.28)	0.93 (0.67, 1.30)
Females	1,202	223	32.49	31.86	0.98 (0.85, 1.13)	0.94 (0.80, 1.10)
<25 years	181	35	4.89	5.00	1.02 (0.69, 1.47)	1.50 (0.69, 3.26)
25–64 years	828	148	22.38	21.14	0.94 (0.79, 1.13)	0.95 (0.74, 1.20)
65+ years	193	40	5.22	5.71	1.10 (0.76, 1.55)	0.74 (0.44, 1.24)
RELATIONSHIP BREAKDOWN						
All	1,145	191	30.95	27.29	0.88 (0.75, 1.03)	0.97 (0.78, 1.19)
Males	923	152	24.95	21.71	0.87 (0.73, 1.03)	0.84 (0.57, 1.25)
<25 years	104	23	2.81	3.29	1.17 (0.71, 1.85)	2.24 (0.85, 5.90)
25–64 years	758	118	20.49	16.86	0.82 (0.67, 0.99)^**^	0.78 (0.48, 1.25)
65+ years	61	11	1.65	1.57	0.95 (0.45, 1.83)	0.31 (0.05, 1.84)
Females	222	39	6.00	5.57	0.93 (0.64, 1.31)	0.80 (0.56, 1.16)
<25 years	46	7	1.24	1.00	0.80 (0.31, 1.79)	2.09 (0.35, 12.41)
25–64 years	160	31	4.32	4.43	1.02 (0.67, 1.51)	0.90 (0.55, 1.47)
65+ years	16	<5	0.43	a	0.33 (0.01, 2.13)	0.49 (0.02, 12.62)
FINANCIAL STRESSORS						
All	369	73	9.97	10.43	1.05 (0.80, 1.35)	1.43 (0.77, 2.64)
Males	312	66	8.43	9.43	1.12 (0.84, 1.46)	1.36 (0.76, 2.45)
<25 years	19	6	0.51	0.86	1.67 (0.55, 4.35)	3.27 (0.93, 11.45)
25–64 years	265	51	7.16	7.29	1.02 (0.74, 1.38)	1.37 (0.73, 2.58)
65+ years	28	9	0.76	1.29	1.70 (0.71, 3.70)	2.88 (0.98, 8.44)
Females	57	7	1.54	1.00	0.65 (0.25, 1.43)	1.95 (0.24, 15.97)
<25 years	<5	0	a	0.00	0 (0, 28.14)	b
25–64 years	48	6	1.30	0.86	0.66 (0.23, 1.55)	2.51 (0.28, 22.39)
65+ years	7	<5	0.19	a	0.76 (0.02, 5.88)	1.52 (0.10, 22.65)
UNEMPLOYMENT						
All	280	81	7.57	11.57	1.53 (1.18, 1.96)^**^	1.26 (0.70, 2.26)
Males	237	71	6.41	10.14	1.58 (1.20, 2.07)^**^	1.16 (0.63, 2.12)
<25 years	25	9	0.68	1.29	1.90 (0.78, 4.21)	1.68 (0.35, 7.99)
25–64 years	204	59	5.51	8.43	1.53 (1.12, 2.05)^**^	1.07 (0.52, 2.21)
65+ years	8	<5	0.22	a	1.98 (0.34, 8.26)	0.74 (0.18, 3.06)
Females	43	10	1.16	1.43	1.23 (0.55, 2.48)	1.42 (0.57, 3.55)
<25 years	<5	0	a	0.00	0 (0, 28.14)	b
25–64 years	39	10	1.05	1.43	1.36 (0.6, 2.76)	1.58 (0.61, 4.07)
65+ years	<5	0	a	0.00	0 (0, 28.14)	b
HOMELESSNESS						
All	75	15	2.03	2.14	1.06 (0.56, 1.86)	0.54 (0.29, 1.01)
Males	61	13	1.65	1.86	1.13 (0.57, 2.07)	0.57 (0.28, 1.16)
<25 years	<5	<5	a	a	3.52 (0.29, 30.76)	b
25–64 years	52	11	1.41	1.57	1.12 (0.53, 2.17)	0.52 (0.24, 1.13)
65+ years	6	0	0.16	0.00	0 (0, 4.49)	b
Females	14	<5	0.38	a	0.76 (0.08, 3.29)	0.43 (0.08, 2.27)
<25 years	<5	<5	a	a	2.64 (0.04, 50.77)	b
25–64 years	10	0	0.27	0.00	0 (0, 2.36)	b
65+ years	<5	<5	a	a	2.64 (0.04, 50.77)	b

*a, not shown due to low cell count; b, model could not be fitted to the data*.

** *P < 0.05*.

Considering risk factors, the unadjusted analysis showed significant differences in suicide in the context of unemployment and relationship breakdown during the COVID-19 period compared to the pre-COVID-19 period. Analysis showed an increase in the number of suicides occurring in the context of unemployment in the COVID-19 period (unadjusted RR 1.53 [95% CI 1.18–1.96]). Specifically, there were increases in suicides in which unemployment was implicated in males (unadjusted RR 1.58 [95% CI 1.20–2.07]) and in males aged 25–64 years (unadjusted RR 1.53 [95% CI 1.12–2.05]). In contrast, there was a decrease in the number of suicides occurring in the context of relationship breakdown in the COVID-19 period (unadjusted RR 0.82 [95% CI 0.67–0.99]). However, no significant changes related to risk factors remained after adjusting the models.

## Discussion

Through analysis of real-time suicide surveillance data in Queensland, Victorian and Tasmania, we found no overall increase in suicides in the initial seven months of the pandemic compared to the pre-COVID-19 period. However, we identified an increase in suicides in young males. Additionally, unemployment was a stressor implicated in more suicides occurring during the COVID-19 period compared to the pre-COVID-19 period; this was accounted for by an increase in these cases in males aged 25–64 years. There was no increase in unemployment-related suicides in young males despite this age group showing an overall increase in the number of suicides over the initial months of the pandemic. We also identified a small decrease in suicides in men aged 25–64 years occurring in the context of relationship breakdown. Although these changes in suicides in the context of unemployment and relationship breakdown were identified in our unadjusted analysis, no significant changes were identified in our adjusted analysis. As such, we have only low to moderate confidence in these findings since they were not adjusted for typical confounding variables in time series analyses. The finding we have the most confidence in is the significant increase in suicides in young males in the first 7 months of the COVID-19 pandemic.

Our finding of no overall increase in suicides is consistent with those of other published studies from high-income and upper-middle-income countries, which have found either decreases or no changes in suicide rates over the early months of the pandemic (6–8, 16–18). The largest of these studies analyzed data from 21 countries (including all three data sources used in our study) and found no evidence of a significant increase in suicide rates since the beginning of the pandemic to the end of July 2020 (6). The circumstances the pandemic created could have had positive impacts on mental health, especially in the initial months of the pandemic (6, 19). It is therefore possible that some positive impacts of COVID-19 may have balanced or served as protective against the expected negative impacts and could at least partially explain why we did not find an overall increase in suicides in the early months of the pandemic.

Given these previous studies, it is unsurprising that we found no overall increase in suicides in the initial months of the pandemic. However, pooling data from three registers enabled stratified analyses by sex and age group and identified a significant increase in suicides in males aged younger than 25 years. There is considerable research regarding adverse outcomes associated with the pandemic that suggests young people are disproportionately affected (20, 21). A recent study of suicide rates found that effects of the pandemic were unevenly distributed across populations in Japan (10). Although the greatest increases in suicides in Japan were observed in females rather than in males, as we found, significant increases were also identified in people aged younger than 20 years (10). Young males warrant special consideration as a group that may be particularly adversely affected by the pandemic. We know that rates of suicide in this group are already high (22), and that certain factors may contribute to their over-representation in suicide statistics. For example, compared with their female counterparts, males are more likely to choose lethal means (23), more likely to use drugs and alcohol (24), and less likely to seek help (24). These factors, combined with the enormity of the mental health and economic impacts of COVID-19, may mean that the pandemic has particularly affected young males.

Despite young males being the only age group to show an overall increase in suicides, we could not directly ascertain whether any of the four risk factors appeared to be contributing to this increase. However, a few findings from our adjusted analysis may give us a clue as to what might be driving this increase in young men. After adjustment for confounding, we observed large rate ratios for young men in the context of relationship breakdown and financial difficulties (RR = 2.24 and 3.27, respectively). These rate ratios, however, were non-significant, and this is likely due to a lack of statistical power. If data were available for the whole country, or for a more extended period, it is conceivable that these findings would be statistically significant.

It is also possible that other risk factors contributed to the observed increase in suicides in young men. It was beyond the scope of this study to investigate all potential risk factors that might have been heightened by the pandemic, although young people might have been particularly affected by measures that were necessarily introduced to reduce the spread of COVID-19 such as lockdowns. These measures have increased isolation (4), a known risk factor for suicide (25). In addition, young people's educational experiences have been greatly impacted during the pandemic and uncertainty about future employment is likely given the current circumstances. Continued monitoring of the overall number of suicides and risk factors associated with suicides in young men is essential.

We found some evidence of an increase in the number of suicides occurring in the context of unemployment, largely accounted for by an increase in these suicides in males aged 25–64 years. This is consistent with research that showed increases in suicide rates in most countries that were affected by the 2008 Global Economic Crisis, particularly for men affected by unemployment (12). The identified increase in unemployment-related suicides in our study occurred in the early months of the pandemic despite the introduction or strengthening of financial supports. The Australian Government funded initiatives (e.g., JobKeeper and JobSeeker) sought to buffer the effects of unemployment and underemployment as a result of the pandemic (5). Early results from the “Taking the Pulse of the Nation” survey of 1,200 adults suggest that following these government initiatives, self-reported financial stress fell to 20% (from 25% in early months of the pandemic) and remained steady during June and July 2020 (5). However, in the second half of 2020, with the announcement of plans to reduce support, financial stress increased even though the economy was starting to re-open (5). It will be critical to continue to monitor the impact of unemployment and financial stressors on suicide, especially as these government supports are scaled back.

In contrast to expectations, we found a slight decrease in suicides in men aged 25–64 years occurring in the context of relationship breakdown. It is possible that some relationships may have strengthened because couples and families spent more time together, which could protect against suicide. For others, relationship difficulties may have been significant and even exacerbated pre-existing difficulties, but couples may have been unable to separate (especially in areas that had protracted lockdowns) which may have artificially masked relationship breakdowns. There is some evidence of a decrease in divorce rates in the US during the early months of the pandemic but in some states initial declines have now rebounded (26).

### Implications

Continued monitoring of suicide data is essential, particularly in the context of roll-backs of financial supports and implementation of further lockdowns. This monitoring should stratify by key demographic subgroups, and should also continue to consider risk factors such as unemployment. This monitoring is especially crucial given that in the Australian context there is some evidence that after initial reductions in emergency mental health-related ambulance call-outs and presentations to emergency departments (27–29), increases have occurred in the later months of the pandemic (29). Ideally, patterns of suicide in different geographical areas and different populations could be examined, not only at the state level but in smaller geographical regions and in populations that may be disproportionately affected by the impacts of the pandemic. It was beyond the scope of this analysis to consider external factors that might have influenced suicide patterns in different states, including varying public health measures or economic support packages, but this could also be an area for future research.

While we found no significant increase in the number of suicides occurring in the context of financial or relationships stressors during COVID-19, Queensland (7) and Victorian (8) research has explored the COVID-19 context in individual suicides. One consistent finding was that financial situations and interpersonal relationships were notable domains in which stress manifested in suicides that were considered COVID-19 related. This suggests that, even if COVID-19 did not significantly affect the prevalence of these stressors, it may impact how individuals experience these stressors, which may be a valuable area for further exploration.

Qualitative research, particularly with people with lived experiences of suicide, is essential to understand the stories behind the statistics presented in this study. The increase in suicides in young males is worrying and given none of the stressors we studied appeared to be more commonly reported by police in suicides by this group during the pandemic, further research focusing on young males is essential.

### Strengths and Limitations

This study had several strengths. Firstly, we combined data from Queensland, Victoria and Tasmania, enabling us to include approximately half of all Australian suicides that would have occurred over the study period. In addition, using this register data allowed us to examine trends in a way that would not have been possible had we relied on official suicide statistics. The data we drew upon are being produced in near real-time in these three states, and other states have or are also now establishing similar registers. This creates the opportunity to extend the analysis into the present and produce “rolling updates” on the themes and issues identified here.

Against this, the study also had several limitations. One limitation is the reliance on information provided by police. In Queensland and Tasmania, the standardized police forms used for this study were completed by a police officer soon after death, and in Victoria we used the information from the initial police summary of circumstances. The police summaries of circumstances vary greatly in detail, and the reliance on information supplied by police very soon after the death in all data sources, introduces the possibility of misclassification bias, like underreporting risk factors. In contrast, most previous research using the suicide registers used data contained in the full suicide registers. In Victoria for example, this means the entire coronial file including the coronial brief, forensic medical and scientific reports (i.e., autopsy and toxicology reports), and coroners' findings are typically available to coders (30). The presence of recent relationship breakdown reported in our study is largely consistent with research published using the full suicide registers in Victoria (31) and Queensland (32). However, the presence of other risk factors such as unemployment and financial related stressors is lower in this study compared to another Victorian study (33). Another related limitation is that we could only examine risk factors consistently captured across the three suicide registers and that could be reasonably expected to be recorded by the police (i.e., known about at the time of initial police investigations). In addition, the number of monthly suicides was often low, meaning that small changes could result in large rate ratios, even though the absolute change was small. Our study may be underpowered because statistical power in this context is largely a function of the number of time points available for analysis, with optimal power achieved when there are large numbers of time points in the periods of interest. Our study had seven months available for analysis for the COVID-19 period and 37 from the pre-COVID period. However, the need to use data with high statistical power must be balanced against the broader need to identify trends that signify worsening mental health conditions and, therefore an increased suicide risk.

## Conclusion

Our findings reinforce the importance of proactive responses to the mental health and economic consequences of the pandemic. Although our analysis found no evidence of an overall increase in suicides after the pandemic began, the picture is complex. The identified increase in suicide in young men indicates that the impact of the pandemic is likely unevenly distributed across populations. The increase in suicides in the context of unemployment reinforces the strong need for mitigation measures during COVID-19, and for ongoing monitoring of suicide as the pandemic continues.

## Data Availability Statement

The datasets presented in this article are not readily available because of data confidentiality agreements. Requests to access the datasets should be directed to Angela Clapperton, angela. clapperton@unimelb.edu.au.

## Ethics Statement

The studies involving human participants were reviewed and approved by the University of Melbourne's Human Research Ethics Committee (Reference Number: 2021-21322-16795-3). Written informed consent from the participants' legal guardian/next of kin was not required to participate in this study in accordance with the national legislation and the institutional requirements.

## Author Contributions

JP, JD, KK, SL, AG, AC, and MS designed the study. JD, AG, KK, SL, and CM collected the data. AC and MS analyzed the data. AC wrote the first draft of the manuscript. All authors contributed to the interpretation of the findings and reviewed and revised drafts of the manuscript.

## Funding

This work was funded by the Australian Institute of Health and Welfare (AIHW).

## Conflict of Interest

The authors declare that the research was conducted in the absence of any commercial or financial relationships that could be construed as a potential conflict of interest.

## Publisher's Note

All claims expressed in this article are solely those of the authors and do not necessarily represent those of their affiliated organizations, or those of the publisher, the editors and the reviewers. Any product that may be evaluated in this article, or claim that may be made by its manufacturer, is not guaranteed or endorsed by the publisher.

## References

[1] Woolf SH Chapman DA Lee JH. COVID-19 as the leading cause of death in the United States. JAMA. (2021) 325:123–4. 10.1001/jama.2020.24865 33331845 PMC8553021

[2] McGorry P. Mental health and COVID-19: are we really all in this together? Med J Aust. (2020) 213:454–5. 10.5694/mja2.50834 33107074

[3] John A Okolie C Eyles E Webb RT Schmidt L McGuiness LA. The impact of the COVID-19 pandemic on self-harm and suicidal behaviour: a living systematic review. F1000Res. (2020) 9:1097. 10.12688/f1000research.25522.1 33604025 PMC7871358

[4] Gunnell D Appleby L Arensman E Hawton K John A Kapur N. Suicide risk and prevention during the COVID-19 pandemic. The Lancet Psychiatry. (2020) 7:468–71. 10.1016/S2215-0366(20)30171-1 32330430 PMC7173821

[5] Botha F Butterworth P Wilkins R. “Heightened mental distress: can addressing financial distress help?,” In: Broadway B, Payne A, Salamanca N, editors. Coping with COVID-19: Rethinking Australia. Melbourne: Melbourne Institute (2020). p. 13–6.

[6] Pirkis J John A Shin S DelPozo-Banos M Arya V Analuisa-Aguilar P. Suicide trends in the early months of the COVID-19 pandemic: an interrupted time-series analysis of preliminary data from 21 countries. The Lancet Psychiatry. (2021) 8, 579–88. 10.1016/S2215-0366(21)00091-2 33862016 PMC9188435

[7] Leske S Kõlves K Crompton D Arensman E de Leo D. Real-time suicide mortality data from police reports in Queensland, Australia, during the COVID-19 pandemic: an interrupted time-series analysis. The Lancet Psychiatry. (2020) 8, 58–63. 10.1016/S2215-0366(20)30435-1 33212023 PMC7836943

[8] Dwyer J Dwyer J Hiscock R O'Callaghan C Taylor K Millar C. COVID-19 as a context in suicide: early insights from Victoria, Australia. Aust N Z J Public Health. (2021). 10.1111/1753-6405.13132 34251732 PMC8441721

[9] John A Pirkis J Gunnell D Appleby L Morrissey J. Trends in suicide during the covid-19 pandemic. BMJ. (2020) 371:m4352. 10.1136/bmj.m4352 33184048

[10] Tanaka T Okamoto S. Increase in suicide following an initial decline during the COVID-19 pandemic in Japan. Nat Hum Behav. (2021) 5:229–38. 10.1038/s41562-020-01042-z 33452498

[11] Nomura S Kawashima T Yoneoka D Tanoue Y Eguchi A Gilmour S. Trends in suicide in Japan by gender during the COVID-19 pandemic, up to September 2020. Psychiatry Res. (2021) 295:113622. 10.1016/j.psychres.2020.113622 33290942

[12] Chang SS Stuckler D Yip P Gunnell D. Impact of 2008 global economic crisis on suicide: time trend study in 54 countries. BMJ. (2013) 347:f5239. 10.1136/bmj.f5239 24046155 PMC3776046

[13] Australian Government Department of Health. First *Confirmed Case of Novel Coronavirus in Australia*. (2020). Available online at: https://www.health.gov.au/ministers/the-hon-greg-hunt-mp/media/first-confirmed-case-of-novel-coronavirus-in-australia. (accessed June 20, 2021).

[14] Australian Government Department of Health. Update on Novel Coronavirus. (2020). Available online at: https://www.health.gov.au/ministers/the-hon-greg-hunt-mp/media/update-on-novel-coronavirus (accessed February 15, 2020).

[15] Leske S Adam G Schrader I Catakovic A Weir B Crompton D. “Suicide in Queensland: Annual Report 2020,” In: Australian Institute for Suicide Research and Prevention: School of Applied Psychology, Griffith University (2020).31732765

[16] Calderon-Anyosa RJC Kaufman JS. Impact of COVID-19 lockdown policy on homicide, suicide, and motor vehicle deaths in Peru. Prev Med. (2021) 143:106331. 10.1016/j.ypmed.2020.106331 33232687 PMC7680039

[17] Vandoros S Theodorikakou O Katsadoros K Zafeiropoulou D Kawachi I. No evidence of increase in suicide in Greece during the first wave of Covid-19. medRxiv [Preprint]. (2020). 10.1101/2020.11.13.20231571

[18] Faust JS Shah SB Du C Li SX Lin Z Krumholz HM. Suicide Deaths During the COVID-19 Stay-at-Home Advisory in Massachusetts, March to May 2020. JAMA Netw Open. (2021) 4:e2034273. 10.1001/jamanetworkopen.2020.34273 33475750 PMC7821026

[19] Wasserman D Iosue M Wuestefeld A Carli V. Adaptation of evidence-based suicide prevention strategies during and after the COVID-19 pandemic. World Psychiatry. (2020) 19:294–306. 10.1002/wps.20801 32931107 PMC7491639

[20] Yard E Radhakrishnan L Ballesteros MF Sheppard M Gates A Stein Z. Emergency Department Visits for Suspected Suicide Attempts Among Persons Aged 12–25 Years Before and During the COVID-19 Pandemic — United States, January 2019–May 2021. Morbid Mortal Wkly Rep. (2021) 70:888–94. 10.15585/mmwr.mm7024e1 34138833 PMC8220953

[21] O'Connor RC Wetherall K Cleare S McClelland H Melson AJ Niedzwiedz CL. Mental health and well-being during the COVID-19 pandemic: longitudinal analyses of adults in the UK COVID-19 Mental Health &amp; Wellbeing study. Br J Psychiatry. (2020) 2020:1–8. 10.31234/osf.io/r8cdw 33081860 PMC7684009

[22] Harrison JE Henley G. “Suicide and hospitalised self-harm in Australia: trends and analysis,” in Injury research and statistics, 93, Cat no INJCAT 169 Canberra: AIHW. (2014).

[23] Elnour A Harrison J. Lethality of suicide methods. Inj Prev. (2007) 14:39–45. 10.1136/ip.2007.016246 18245314

[24] Reavley NJ Cvetkovski S Jorm AF DI. L. Help-seeking for substance use, anxiety and affective disorders among young people: results from the 2007 australian national survey of mental health and wellbeing. Aust N Z J Psychiatry. (2010) 44:729–35. 10.3109/00048671003705458 20636194

[25] Calati R Ferrari C Brittner M Oasi O Olie E Carvalho AF. Suicidal thoughts and behaviors and social isolation: A narrative review of the literature. J Affect Disord. (2019) 245:653–67. 10.1016/j.jad.2018.11.022 30445391

[26] Manning WD Payne KK. Marriage and Divorce Decline during the COVID-19 Pandemic: a case Study of Five States. Socius: Sociol Res Dynamic World. (2021) 7:21. 10.1177/23780231211006976 34307872 PMC8302068

[27] Mitchell RD O'Reilly GM Mitra B Smit V Miller JP Cameron PA. Impact of COVID-19 State of Emergency restrictions on presentations to two Victorian emergency departments. Emerg Med Australas. (2020) 32:1027–33. 10.1111/1742-6723.13606 32748481 PMC7436380

[28] Dragovic M Pascu V Hall T Ingram J Waters F. Emergency department mental health presentations before and during the COVID-19 outbreak in Western Australia. Australas Psychiatry. (2020) 28:627–31. 10.1177/1039856220960673 32961096 PMC7509241

[29] Andrew E Nehme Z Stephenson M Walker T Smith K. The impact of the COVID-19 pandemic on demand for emergency ambulances in Victoria, Australia. Prehosp Emerg Care. (2021) 2021:1–8. 10.1080/10903127.2021.1944409 34152925

[30] Sutherland G Milner A Dwyer J Bugeja L Woodward A Robinson J. Implementation and evaluation of the Victorian Suicide Register. Aust N Z J Public Health. (2018) 42:296–302. 10.1111/1753-6405.12725 29044826

[31] Clapperton A Newstead S Bugeja L Pirkis J. Relative risk of suicide following exposure to recent stressors, Victoria, Australia. Aust N Z J Public Health. (2019) 43:254–60. 10.1111/1753-6405.12886 30830716

[32] Kolves K Potts B De Leo D. Ten years of suicide mortality in Australia: socio-economic and psychiatric factors in Queensland. J Forensic Leg Med. (2015) 36:136–43. 10.1016/j.jflm.2015.09.012 26454502

[33] Clapperton A Newstead S Bugeja L Pirkis J. Differences in characteristics and exposure to stressors between persons with and without diagnosed mental illness who died by suicide in Victoria, Australia. Crisis. (2018) 40:231–9. 10.1027/0227-5910/a000553 30311798

